# Fire induced reproductive mechanisms of a *Symphoricarpos* (Caprifoliaceae) shrub after dormant season burning

**DOI:** 10.1186/s40529-014-0080-4

**Published:** 2014-12-24

**Authors:** John Derek Scasta, David M Engle, Ryan N Harr, Diane M Debinski

**Affiliations:** 1 Department of Ecosystem Science and Management, University of Wyoming, Laramie, 82071 WY USA; 2 Department of Natural Resource Ecology and Management, Oklahoma State University, Stillwater, OK USA; 3 Iowa Department of Natural Resources, Des Moines, IA USA; 4 Department of Ecology, Evolution, and Organismal Biology, Iowa State University, Ames, IA USA

**Keywords:** Disturbance, Ecology, Prairie, Rangeland, Resprout, Restoration, Woody plant

## Abstract

**Background:**

*Symphoricarpos,* a genus of the Caprifoliaceae family, consists of about 15 species of clonal deciduous shrubs in North America and 1 species endemic to China. In North American tallgrass prairie, *Symphoricarpos orbiculatus* (buckbrush) is the dominant shrub often forming large colonies via sexual and asexual reproductive mechanisms. *Symphoricarpos* shrubs, in particular *S. orbiculatus*, use a unique sexual reproductive mechanism known as layering where vertical stems droop and the tips root upon contact with the soil. Because of conflicting societal values of *S. orbiculatus* for conservation and agriculture and the current attempt to restore historical fire regimes, there is a need for basic research on the biological response of *S. orbiculatus* to anthropogenic burning regimes.

**Results:**

From 2007 through 2013 we applied prescribed fires in the late dormant season on grazed pastures in the Grand River Grasslands of Iowa. From 2011 to 2013, we measured how *S. orbiculatus* basal resprouting and layering stems were affected by patchy fires on grazed pastures, complete pasture fires on grazed pastures or fire exclusion without grazing for more than three years. We measured ramet height, ramet canopy diameter, stems per ramet, ramets per 100 m^2^, and probability of new layering stems 120 days after fire. Height in burned plots was lower than unburned plots but *S. orbiculatus* reached ~ 84% of pre-burn height 120 days after fire. Stems per ramet were 2x greater in the most recently burned plots due to basal re-sprouting. Canopy diameter and density of ramets was not affected by time since fire, but burned pastures had marginally lower densities than plots excluded from fire (*P* = 0.07). Fire triggered new layering stems and no new layering stems were found in plots excluded from fire.

**Conclusions:**

The mechanisms of both basal sprouting and aerial layering after fire suggest *S. orbiculatus* is tolerant to dormant season fires. Furthermore, dormant season fires, regardless if they were patchy fires or complete pasture fires, did not result in mortality of *S. orbiculatus*. Dormant season fires can reduce *S. orbiculatus* structural dominance and maintain lower ramet densities but also trigger basal resprouting and layering.

**Electronic supplementary material:**

The online version of this article (doi:10.1186/s40529-014-0080-4) contains supplementary material, which is available to authorized users.

## Background

Global vegetation distribution patterns are largely driven by climatic constraints and disturbance regimes (Clark [1991]; Collins [2000]). Grasslands on several continents were regulated by the developmental disturbances of fire, large ungulate grazing, and the synergistic interaction of the two (Fuhlendorf and Engle [2001]). The vegetation composition of these fire prone grasslands includes shrubby angiosperm species that persist after fire due to vegetative regeneration mechanisms rather than recruitment (Bond and Midgley [2003]). In North America, conversion to cropland and suppression of fire has rendered tallgrass prairies as one of the most threatened ecosystems globally (Sampson and Knopf [1994]). Prior to European settlement, tallgrass prairie was prone to burning every three to five years (Collins [2000]). Consequently, the exclusion of regular fire in the remaining tallgrass prairie alters plant succession toward a shrub dominated community and shrub encroachment is a global issue (Collins and Adams [1983]; Collins [1992]; Shortt and Vamosi [2012]).

Shrubs in these fire prone ecosystems utilize a resprouting life strategy that results in tradeoffs between growth and reproduction because resprouting shrubs often have smaller seeds, poor reproduction from seeds, and short-lived seed banks (Bond and Wilgen [1996]; Kruger et al. [1997]). The cost of energy allocation belowground to survive fire also reduces growth rate (Bond and van Wilgen [1996]). Subsequently, form follows function, and resprouting species typical of disturbance prone ecosystems are multi-stemmed and short statured to optimize rapid recovery. In contrast, recruiting species are typically single-stemmed and taller, an advantage for the competition for light and dispersion of seed (Bellingham and Sparrow [2000]; Bond and Midgley [2003]). Researchers have focused more on seedling ecology than resprouting mechanisms as a plant life history trait, although neither regeneration strategy is mutually exclusive (Olson and Platt [1995]; Higgins et al. [2000]; Bond and Midgley [2003]). Furthermore, basal resprouting mechanisms are not uniform within a functional group type (i.e., not all shrubs resprout basally or epicormically) or even within genera and are less understood than seedling recruitment (Enright and Goldblum [1999]; Bellingham and Sparrow [2000]; Ansley and Rasmussen [2005]; Winter et al. [2011]).

*Symphoricarpos* is a genus of the honeysuckle or Caprifoliaceae family, consisting of about 15 species of clonal deciduous shrubs in North America and 1 species endemic to China (*S. sinensis*) (Theis et al. [2008])*.* In North America, *Symphoricarpos* is one of the most broadly distributed shrub genera, and *S. orbiculatus* has been described as the dominant shrub of the tallgrass prairie (Holechek et al. [2001]; USDA Plant Database [2013]; Scasta [2014]). Asexual spread is from rhizomes and rooting of aerial stems (henceforth layering) and sexual spread is from seeds (Pelton [1953]; Hullick and Manske [2006]; Nesmith et al. [2006]). Layering occurs when vertical stems droop horizontally and produce adventitious roots when they come into contact with the soil. While the layering mechanism has been studied from a physical disturbance standpoint, it has been largely neglected in the fire ecology literature (Hartmann and Kester [1975]; Deb and Pogener [2012]). Furthermore, *S. orbiculatus* has been characterized as a major invader of unburned prairie but the role of fire exclusion and the application of fire with livestock grazing are not well understood for this species (Bragg and Hulbert [1974]; Stubbendieck et al. [2003]). However, because *S. orbiculatus* is important for a wide range of wildlife species, there are conflicting views on the necessity and desirability of reducing *S. orbiculatus* (Korschgen [1962]; Guthrey [1980]; Brennan [1991]; Soper et al. [1993]; Harrell et al. [2001]; Stubbendieck et al. [2003]).

Quantifying *S. orbiculatus* reproductive mechanisms after fire will yield basic biological insights to assist managers in appropriately applying disturbance regimes and potentially develop alternative strategies to mitigate encroachment. Our objectives were to assess how *S. orbiculatus* vegetative regeneration and reproduction is affected by fire exclusion and late dormant-season fire applied every third year across entire grazed pastures or applied annually on a third of a grazed pasture. Prescribed burning in the late dormant season is the prescribed fire season most common in North American grasslands and is limited to after snow melt and before vegetation greenup. We hypothesized that *S. orbiculatus* is tolerant to dormant season burning and that the reproductive mechanisms of basal sprouting and aerial layering would be dependent on time since fire and serve as the key mechanisms of tolerance. To test our hypothesis we quantified vegetative response to prescribed fire by measuring ramet height, ramet canopy diameter, stems per ramet, ramets per 100 m^2^, and probability of new layering stems.

## Methods

### Study location and design

The study was conducted from 2011–2013 in tallgrass prairie in Ringgold County, Iowa in the Grand River Grasslands (GRG) of Iowa, USA (40°34’N, 94°10’W). The GRG is in the glaciated plains of the Central Tallgrass Prairie Ecoregion and has been described as one of the premier places to restore a functioning tallgrass prairie ecosystem (Missouri Department of Conservation [2010]). Annual precipitation was 968 mm in 2011 (+97 mm above long-term mean), 798 mm in 2012 (− 73 mm below long-term mean), 874 mm in 2013 (+3 mm above long-term mean and averaged 870 mm during the three year study (+1 mm above long-term mean) (Iowa Environmental Mesonet [2014]). Soils are loess hills with glacial till side slopes with slopes in some areas exceeding 9%. Subsoils have high clay content ranging from 42 to 48% and native vegetation was tallgrass prairie (USDA NRCS [2013]). Herbaceous vegetation across all study pastures was dominated by perennial C4 graminoids (*Andropogon gerardii* (big bluestem), *Schizacyrium scoparium* (little bluestem), and *Sorghastrum nutans* (Indiangrass) with a component of exotic C3 graminoids and legumes (*Schedonorus arundinacea* (tall fescue), *Bromus inermis* (smooth brome), *Lotus corniculatus* (birdsfoot trefoil), and *Trifolium spp.* (clover). In these study pastures, *S. orbiculatus* was the most common shrub regardless of the spatial scale of assessment and occurred more often than any other shrub species including *Rhus glabra* (smooth sumac), *Rosa multiflora* (multiflora rose), *Cornus drummondii* (dogwood), *Prunus spp.* (plum) and *Rubus spp.* (raspberry) (Scasta [2014]).

### Fire effects sampling

Three treatments were applied and assessed: 1) Patch-burn grazing (PBG) or burning one-third of a pasture annually (the patch) with cattle having full access to the pasture, 2) Graze and burn (GAB) or burning pastures completely every three years with cattle having full access to the pasture, and 3) Unburned or no fire for over three years and no cattle grazing during that period. Grazing was seasonal from late-April to October with mature Angus cows. Mean (± SE) size of each pasture was 27 ± 3 ha and mean (± SE) stocking rate was 2.3 ± 0.2 AUM ha^−1^. All prescribed fires were conducted within a four week window in the late dormant season (March 8 to April 4) prior to the emergence of *S. orbiculatus* leaves. Mean fire weather data for the available fires was 47% relative humidity, 9 km per hr, and 18°C. The fire and grazing treatments were in place prior to the initiation of sampling in 2011, so all patches and pastures had a consistent elapsed time since fire (0.3, 1, 2 years since fire) (Table 1). The designation of 0.3 years since fire indicates that samples were measured four months after fire. We had less control over the unburned treatments and elapsed time since fire for those pastures was more than three years but the exact number of years is unknown. When we returned in 2013 to conduct a second assessment of unburned pastures, they had all either been burned or mowed so no follow up measurements were possible.

**Table 1:** **Schedule of prescribed fire and measurements of** ***Symphoricarpos orbiculatus*** **in the Grand River Grasslands, USA, 2007-2013**

	Year						
Treatment	2007	2008	2009	2010	2011	2012	2013
**Patch-burn grazed pastures (3 pastures total delineated into 3 patches per pasture)**							
*Pyland North*							
Patch A	fire	-	-	fire	-, m_1_, m_2_	-, m_1_	fire, m_1_, m_2_
Patch B	-	fire	-	-	fire, m_1_, m_2_	-, m_1_	-, m_1_, m_2_
Patch C	-	-	fire	-	-, m_1_, m_2_	fire, m_1_	-, m_1_, m_2_
*Pyland South*							
Patch A	fire	-	fire*	fire	-, m_1_, m_2_	-, m_1_	fire, m_1_, m_2_
Patch B	-	fire	fire*	-	fire, m_1_, m_2_	-, m_1_	-, m_1_, m_2_
Patch C	-	-	fire	-	-, m_1_, m_2_	fire, m1	-, m_1_, m_2_
*Ringgold South*							
Patch A	fire	-	-	fire	-, m_1_, m_2_	-, m_1_	fire, m_1_, m_2_
Patch B	-	fire	-	-	fire, m_1_, m_2_	-, m_1_	-, m_1_, m_2_
Patch C	-	-	fire	-	-, m_1_, m_2_	fire, m_1_	-, m_1_, m_2_
**Graze-and-burn pastures (3 pastures total)**							
*Gilleland*	-	-	fire	-	-, m_1_	fire, m_1_	-, m_1_
*Lee Trail*	-	-	fire	-	-, m_1_	fire, m_1_	-, m_1_
*Pyland West*	-	-	fire	-	-, m_1_	fire, m_1_	-, m_1_
**Unburned pastures (3 pastures total)**							
*Hog Farm*	-	-	-	-	-, m_1_, m_2_	-	-, d
*Kellerton South*	-	-	-	-	-, m_1_, m_2_	-	-, d
*Richardson West*	-	-	-	-	-, m_1_, m_2_	-	-, d

Fire: − = no fire; fire = prescribed fire; fire* = escaped out of the intended burn patch and burned remainder of pasture; d = disrupted (we had less control over the unburned treatment and when we returned in 2013 to measure a second assessment of unburned pastures, they had all either been burned or mowed). (d = disrupted).

*Measurements: m*_*1*_ *= Symphoricarpos orbiculatus maximum ramet height, ramet maximum canopy diameter, stems per ramet measured as number of vertical live stems arising from the same root crown; m*_*2*_ *= new aerial layering stems.*

*Treatments: Patch-burn grazing (PBG) where one-third of a pasture (the patch) is burned and cattle have full access to the pasture, Graze and burn (GAB) where the entire pasture is burned every third year (2012 was the burn year) and cattle have full access to the pasture, and Not burned for more than three years with no grazing. Treatments were in place starting in 2007 so all patches and pastures had a consistent elapsed time since fire (0.3, 1, 2 years since fire).*

**Figure 1:**
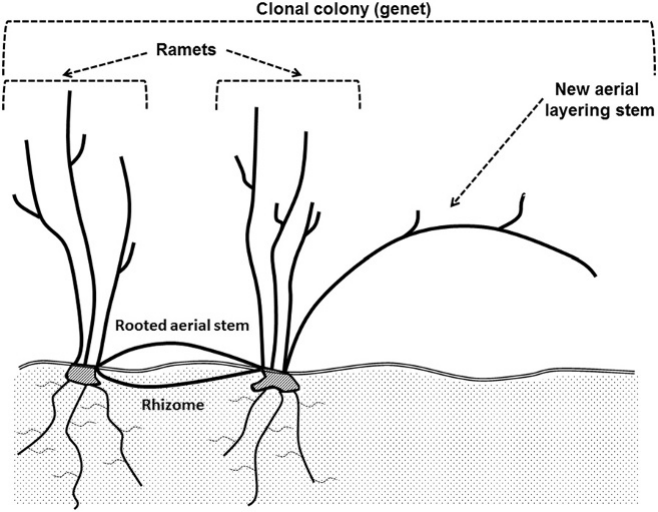
**Diagram of fire effects measurements given the clonal nature of** ***S. orbiculatus*** **.** We conducted measurements at the ramet level and define ramets as individual plants or clonal fragments in the colony that are rooted and may or may not be connected to other ramets by rhizomes or rooted aerial layering stems. Individual ramets were identified by spatial independence based on vertical stems arising from the same root crown. New aerial layering stems were measured as vertical stems arising from the root crown, having a horizontal orientation, and not yet rooting at the tip.

Fire effects measurements were conducted from 2011 to 2013 in six plots per pasture controlling for catena and soil type (Debinski et al. [2011]). Plots were all permanent and ranged from 16 m^2^ (16 m × 1 m) to 350 m^2^ (25 m × 14 m). Plots were variable in size due to the variable density of *S. orbiculatus* ramets. If *S. orbiculatus* was not present within a permanent plot, we assessed 25 m north or south of the permanent marking pins for *S. orbiculatus* presence. Three additional pastures that had not been burned in more than three years were included for fire effects measurements and had three plots per pasture (Table 1). Thus, to ensure adequate sample size and in the sake of time, we sampled plots for a minimum of ten ramets to a maximum of fifty ramets in each plot. Given the clonal nature of *S. orbiculatus* and the intermingling of lateral vegetative structures among adjacent clones, it was not possible to distinguish individual clones (aka, genets). Therefore, we conducted measurements at the ramet level. We define ramets as individual plants or clonal fragments in the colony that are rooted and may have originated from either seed or rooted nodes from aerial layering stems that may or may not be connected to other ramets (Nesmith et al. [2006]) (Figure 1). Ramet measurements were conducted approximately 120 days after fire. Maximum height of the ramet was measured as the tallest individual stem of a ramet. Maximum canopy diameter was measured as the longest horizontal axis of a ramet. Stems per ramet was measured as the total number of vertical live stems arising from the same root crown and is a reflection of the regeneration from basal resprouts. Ramets per 100 m^2^ were measured by noting the total number of ramets in a plot and then converting that to 100 m^2^, a reflection of alterations to ramet density from fire-induced mortality. New aerial layering stems were measured as vertical stems arising from the root crown but having a horizontal orientation but not yet rooting at the tip (Figure 1). All measurements for fire effects were conducted in 2011, 2012 and 2013 except presence/absence of new layering stems (Table 1). New layering stems were only identified in 2011 and 2013 in the PBG pastures and in unburned pastures in 2011 only (Table 1).

### Analysis

To assess the response of *S. orbiculatus* to prescribed fires we used maximum ramet height, maximum ramet canopy diameter and number of live stems per ramet, and density of ramets per 100 m^2^ as response variables. We used plot as a random effect because the plot design accounts for the highly variable topoedaphic features across the landscape, variable disturbance history associated with tillage and over grazing within and across pastures, and soil types. We also used year as a random effect because 2012 was the hottest year since 1895 (for both the mean annual high temperature and the March to May high temperature) and was the 5^th^ driest year since 1895 (based on Palmer’s Drought Severity Index (PDSI) but 2013 was one of the wettest years since 1895 (the 81^st^ driest year based on PDSI) (NOAA [2014]; Wang et al. [2014]). We used mixed effects models (proc mixed) using the residual maximum likelihood estimation method with treatment, elapsed time since fire and the interaction between treatment and elapsed time since fire as fixed effects (SAS Institute [2011]). Covariance parameter estimates for each random effect were calculated and fixed effects assessed for significance (α 0.05). We then conducted post hoc least squares test for all pairwise comparisons of all combinations for fixed effects at the 95% confidence level. Binary presence/absence data of horizontal layering stems data were used to determine odds ratios (odds = *e*^βx^) for the presence/absence probability of layering stems related to elapsed time since fire. The logit function in proc genmod was used to model probability, assess goodness of fit and determine parameter estimate for time since fire (SAS Institute [2011]).

## Results

**Figure 2:**
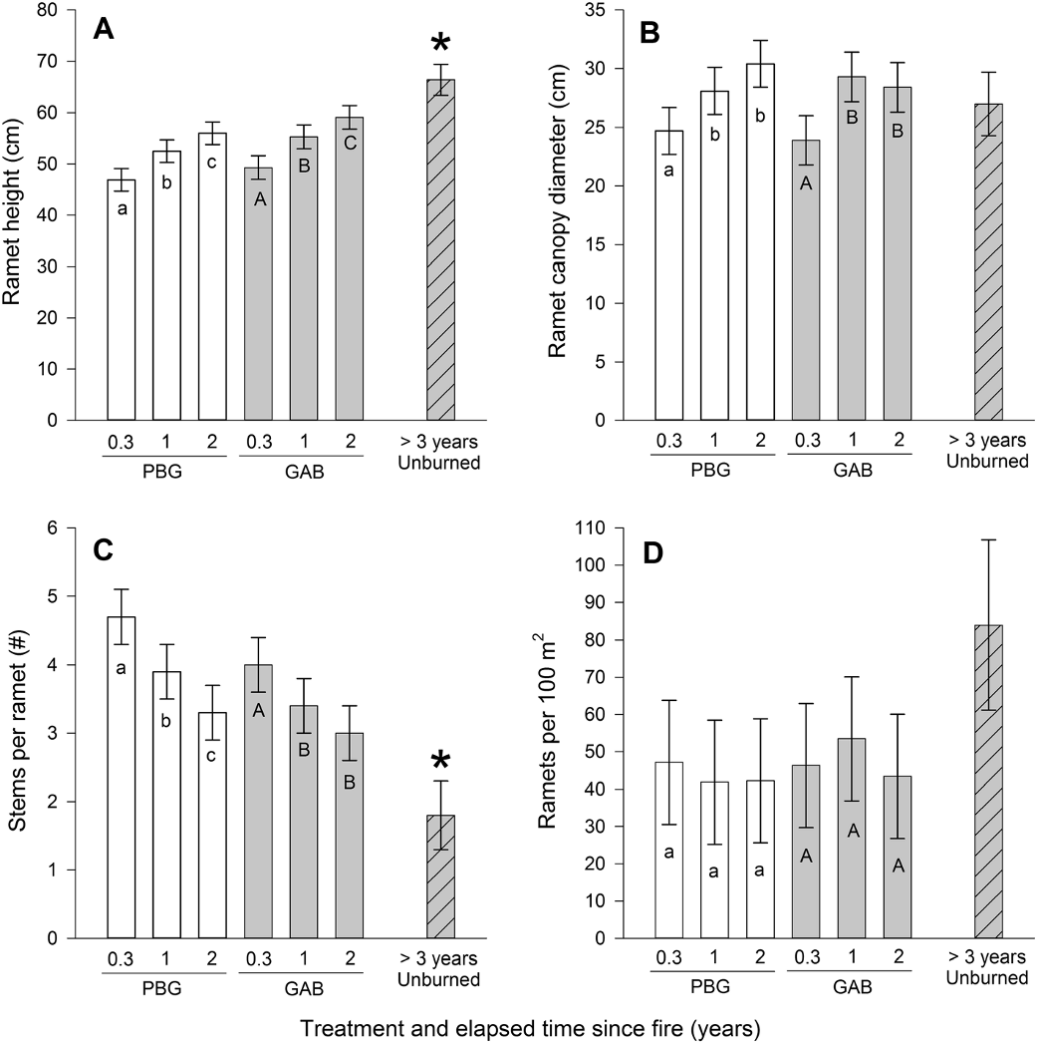
**Mean ± SE of ramet height (A), ramet canopy diameter (B), stems per ramet (C), and ramets per 100 m**^**2**^**(D) for** ***Symphoricarpos orbiculatus*** **plants exposed to three fire treatments in the Grand River Grasslands of Iowa, USA, 2011–2013.** Treatments are 1) Patch-burn grazing (PBG) where one-third of a pasture (the patch) is burned and cattle have full access to the pasture, 2) Graze and burn (GAB) where the entire pasture is burned every third year (2012 was the burn year) and cattle have full access to the pasture, 3) Unburned for greater than three years with no grazing. The *asterisk* indicates if the unburned treatment is significantly different (*P* ≤ 0.05) than the burned treatments and all elapsed time since fire categories (0.3, 1 or 2 years after fire). Letters indicate significant differences within a treatment (*P* ≤ 0.05) (lower case for PBG and capitalized for GAB) based on a mixed effects model.

Ramet height on burned pastures, regardless of how fire was applied (henceforth, whether fire was applied PBG or GAB), was significantly lower (*F*_2,66_ = 5.47; *P* < 0.01) than pastures managed without fire. Ultimately, the lower ramet height on burned pastures is largely a function of elapsed time since fire rather than how fire was applied because time since fire was positively correlated with ramet height (*F*_3,64_ = 23.66; *P* < 0.01) and the two burned treatments did not differ (*P* = 0.32) (Figure 2A). The tallest *S. orbiculatus* ramets measured were 114 cm and were found in unburned pastures or GAB pastures 2 years after fire. Four months after fire, ramet height had reached ~ 84% of its pre-fire height, regardless of how fire was applied.

Ramet canopy diameter did not differ between the three treatments (*F*_2,66_ = 0.09; *P* = 0.92) (Figure 2B). However, the interaction between treatment and elapsed time since fire was significant (*F*_6,62_ = 4.74; *P* < 0.01) as canopy diameter within PBG and GAB treatments, respectively, was lower at 0.3 years after fire than 1 or 2 years after fire (Figure 2B). The greatest ramet canopy diameter measured was 143 cm and was in a GAB pasture 2 years after fire.

Ramets in the most recently burned patches in PBG pastures had 2.6 times more stems, and ramets in GAB pastures the year it was burned had 2.2 times more stems than pastures not burned in over 3 years. The number of stems per ramet on burned pastures, regardless of how fire was applied, was significantly higher (*F*_2,66_ = 6.39; *P* < 0.01) than pastures not burned in over 3 years (Figure 2C). Elapsed time since fire was negatively correlated with stems per ramet (*F*_3,64_ = 17.79; *P* < 0.01) and the two burned treatments did not differ (*P* = 0.30) (Figure 2C). Thus, the relationship between number of stems per ramet and fire is largely a function of elapsed time since fire rather than how fire was applied.

Ramets per 100 m^2^ did not differ between the three treatments (F_2,66_ = 0.03; *P* = 0.30). However, the interaction between treatment and elapsed time since fire was marginally significant (F_6, 62_ = 2.04; *P* = 0.07) with PBG and GAB treatments displaying consistently lower densities than unburned pastures (Figure 2D). There was no apparent mortality in recently burned PBG patches or when complete GAB pastures were burned (Figure 2D).

**Figure 3:**
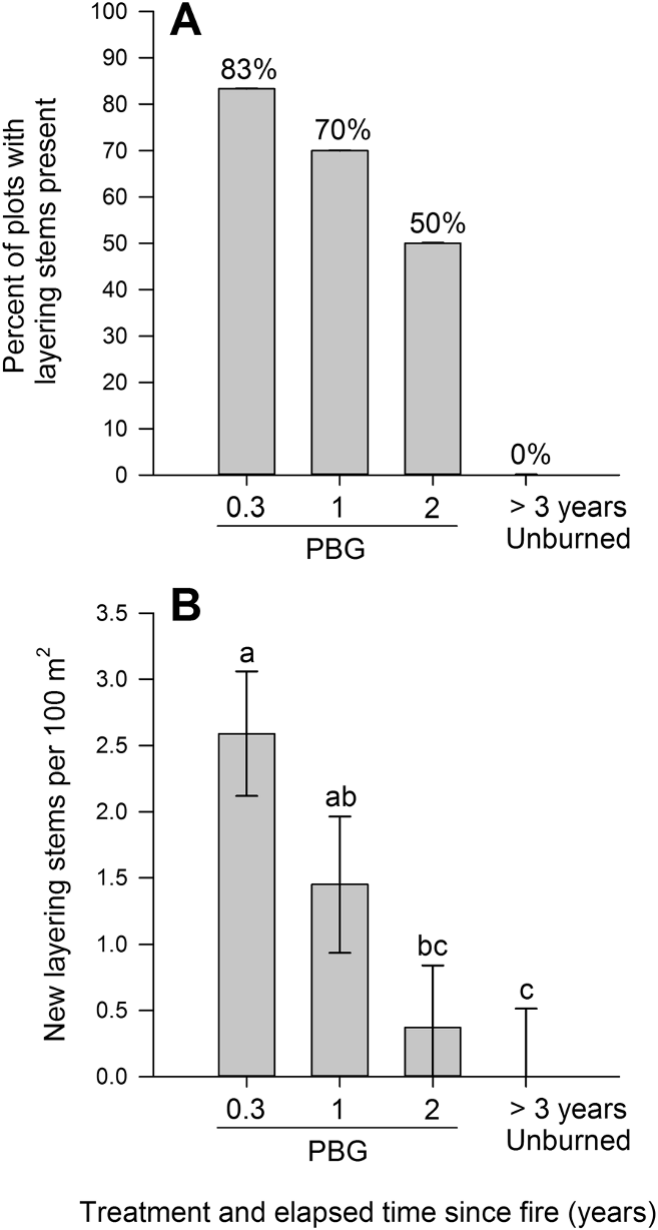
**Effect of elapsed time since fire on new** ***Symphoricarpos orbiculatus*** **horizontal layering stems based on percent of plots with new layering stems (A), and new layering stem density (B) in the unburned pastures and the Patch-burn grazed (PBG) pastures in in the Grand River Grasslands of Iowa, USA, 2011 and 2013.** Letters indicate differences (*P* ≤ 0.05) based on a mixed effects model.

Using logistic regression of the binomial presence/absence of new layering stems, the parameter estimate for elapsed time since fire was −1.3 and was significant (*P* < 0.01) (Table 2). Based on the exponentiation of the parameter estimate, the odds of having a layering stem present in a plot improve 3.77 times for every year closer to the burn year. Therefore, 83% of the most recently burned patches had layering stems present with a steady decrease as time since fire elapsed. Pastures not burned in over 3 years had no new layering stems (*P* < 0.01) (Figure 3A). In a similar fashion, as time since fire elapsed, the density of layering stems per 100 m^2^ declines (*P* < 0.01) (Figure 3B).

**Table 2:** **Maximum likelihood estimates for time since fire and probability of new** ***Symphoricarpos orbiculatus*** **layering stems**

Parameter	Estimate	Standard	Likelihood ratio 95%		***P***value
		Error	Confidence limits		
Intercept	3.420	1.077	1.537	5.858	0.002
Time Since Fire	−1.330	0.403	−2.235	−0.622	0.001

*Maximum likelihood parameter estimates for time since fire and probability of the presence of new aerial layering stems based on the logit function and binomial presence/absence data. Based on the exponentiation of the parameter estimate, the odds of having a layering stem present improve 3.77x for every year closer to the burn year.*

## Discussion

Our study examining the regeneration and reproduction of *S. orbiculatus* after fire indicates that this shrub is tolerant of low-intensity prescribed fires conducted in the late dormant season or early spring prior to emergence of leaves. *S. orbiculatus* rapidly regenerated from basal resprouts, recovering to ~ 84% of its pre-fire height 120 days after fire with no apparent mortality. *S. orbiculatus* used both basal resprouting and aerial layering as reproduction strategies to recover after the disturbance of fire. Ramet height increased with time since fire, but stems per ramet and aerial layering probability decreased with time since fire. Initially, fire physically altered shrub structure as measured by ramet height and stems per ramet. However, the initial structural change was only temporary as the trend indicated that as time elapses past four years, physical shrub structure becomes similar to unburned pastures, similar to *Artemisia filifolia* (sand sagebrush) (Winter et al. [2011]).

The rapid structural regeneration of *S. orbiculatus* within the burn-year growing season is similar to other species that display rapid regrowth the first year with a declining rate of growth in the next three to four years (Gratani and Amadori [1991]). The rate of recovery for *S. orbiculatus* was slower than *Prosopis glandulosa* (mesquite) that recovered to pre-burn heights within one year after fire (Drewa [2003]). A similar study in tallgrass prairie suggested that *R. glabra* rapidly recovers to its pre-burn height and fire can result in greater plant heights than in unburned areas for both *P. glandulosa* and *R. glabra* (Drewa [2003]; Hajny et al. [2011]). However, fire did not appear to increase *S. orbiculatus* height in our study. In more arid environments with coarse sandy soils, *A. filifolia* and *Quercus havardii* (sand shinnery oak) recovered to only ~ 55% of pre-burn height within the first year of a burn (Harrell et al. [2001]; Winter et al. [2011]). However, *S. orbiculatus* is similar to *A. filifolia* and *Q. havardii* because it took three years or more for all three species to recover to pre-burn heights. Considering these variable responses, fire only altered the physical structure of these fire-tolerant shrubs for a relatively short period, typically less than four years, and these shrubs regenerated at different rates depending on climate and soil limitations (Olson and Platt [1995]; Iwasa and Kubo [1997]).

Because *S. orbiculatus* seeds have little to no potential to form a long lived soil seed bank (Hidayata et al. [2001]) and the rapid regeneration after fire reported herein, vegetative regeneration appears to be the primary recovery mechanism of *S. orbiculatus* to dormant season prescribed fires. In context, the tallgrass prairie is a disturbance driven ecosystem that historically had a fire return interval of three to five years (Collins [2000]). The life history tradeoff between resprouting and seedling recruitment is largely determined by disturbance regimes and tallgrass prairie was historically disturbed regularly by fire (Wells [1969]; Bellingham and Sparrow [2000]; Collins [2000]). Episodic resprouting events triggered by fire are followed by extended periods of self-thinning as indicated by the relationship with elapsing time since fire and declining stems per ramet in our study (Clark [1991]; Hodgkinson [1998]).

The low growing and multi-stemmed architecture of *S. orbiculatus* is also reflective of the disturbance regime of tallgrass prairie and its ability to rapidly recover after fire (Midgley [1996]; Bellingham and Sparrow [2000]). However, vegetative reproduction of *S. orbiculatus* after fire is not limited to basal resprouting regeneration but also appears to include aerial layering. The initiation of aerial layering has also been reported to be triggered by physical disturbances for *Rubus trivialis* (dewberries) from grazing, for *Oplopana horridus* (devil’s club) from recent logging activity, and for *Cercocarpus* spp. (mountain mahogany) from grazing and possibly fire (Miller [1964]; Abrahamson [1975]; Lantz and Antos [2002]). The absence of cattle grazing activity as a source of physical disturbance in the unburned pastures could also help explain the lack of new *S. orbiculatus* layering stems (Abrahamson [1975]). In the Douglas-Fir forests of Oregon, USA, burning did not increase aerial layering but did increase basal resprouting of *Acer circinatum* (vine maple) while the physical disturbance of thinning and falling branches stimulated layering (O’Dea et al. [1995]). However, in this old growth forest ecosystem the historic fire return interval is estimated to be 230 years compared to our study area that burned every three to five years (Agee [1993]).

Our results quantifying the ability of *S. orbiculatus* to regenerate after low-intensity prescribed fires conducted in the late dormant season or early spring is similar to other *Symphoricarpos* species, although not every species is noted to be tolerant of fire. *S. occidentalis* (western snowberry) and *S. albus* (common snowberry) are highly tolerant to fire due to vegetative regeneration mechanisms (Mclean [1969]; Anderson and Bailey [1979]; Morgan and Neuenschwander [1988]; Romo et al. [1993]; Youngblood et al. [2006]; Hauser [2007]). In contrast, *S. oreophilus* (mountain snowberry), *S. longiflorus* (longflower snowberry), and *S. mollis* (creeping snowberry) have been reported to be only low to moderately resilient to fire (Bartos and Mueggler [1982]; Snyder [1991]; Aleksoff [1999]; McWilliams [2000]; McWilliams [2005]; Knapp et al. [2006]; Rocca [2009]). Interestingly, even if a species is not highly tolerant of fire, it still may be fire dependent as *S. oreophilus* occurred only on burned sites (Poreda and Wullstein [1994]). This variable response to fire within the genus is attributed to species distributions and biogeographic disturbance patterns because disturbance frequency is a major determinant of resprouting strength (Westman and O’Leary [1986]; USDA Plant Database [2013]).

In contrast to our study, other studies have reported reductions in *S. orbiculatus* with fire. Late spring burning in tallgrass prairie reduced *S. orbiculatus* due to lower carbohydrate reserves because plants had already leafed out (Hulbert [1984]) which is different than our early spring burns and no apparent reductions. Furthermore, two successive years of spring burning in April in a forested corridor of Kansas reduced *S. orbiculatus* canopy cover from 40% to 9% but did not alter shrub species richness (Abrams [1988]). Thus, burning later in the season or burning a site repeatedly for successive years may result in reductions of *S. orbiculatus*. However, at our research sites, most burning is done in the early spring and may not be possible in late spring due to *Schedonorus arundinaceus* (tall fescue) greenup and alteration of fire behavior (McGranahan et al. [2012]). Furthermore, it can be difficult to graze and burn complete pastures for successive years (Ansley et al. [2010]).

Woody plant encroachment in mesic grasslands was regulated by fire (Pyne [1997]; Briggs et al. [2005]). However, fire has largely been removed from the landscape or is only applied in a short seasonal window in the late-dormant season leading to the need for herbicide treatments (Smith [1977]; Defelice [1991]; Stubbendieck et al. [2003]). It is important to consider that historically wildland fire seasonality, intensity and frequency may have been very different than conventional applications of prescribed fire. Our study suggests that a three-year fire return interval using dormant season prescribed fires does not result in plant mortality but can result in altered physical structure and could maintain lower densities of *S. orbiculatus*. While our study did not document mortality, this is a common result for resprouting shrubs (Canadell et al. [1991]; Olson and Platt [1995]). However, we did document lower densities on pastures managed with regular fire compared to higher densities on pastures managed with long-term fire exclusion but the stimulation of aerial layering by fire could theoretically increase density. This contradiction needs additional research across greater temporal scales. Furthermore, unlike other shrub species such as *R. glabra* that may increase with fire, *S. orbiculatus* density and expansion appears to be hindered by regular fire but further research is needed on other seasonal applications and intensities of fire (Hajny et al. [2011]).

Our study also included the spatio-temporal application of patchy fires (PBG) and cattle grazing, an attempt to recouple pre-settlement fire and grazing processes (Burrows [1991]; Fuhlendorf and Engle [2001]). The attraction to recently burned patches alters grazing patterns and results in fuel accumulation in long-unburned patches and potentially enhances fire behavior (Kerby et al. [2007]). However, when compared to attempting to burn a pasture completely, we did not observe different vegetative responses associated with fire intensity or herbivory. This is not surprising because *S. orbiculatus* is an extremely effective resprouter after fire and cattle do not graze *S. orbiculatus* (Stubbendieck et al. [2003]).

## Conclusion

Our documentation of fire triggering aerial layering of *S. orbiculatus* is a new insight relevant to fire-prone mesic grasslands. Other studies have documented physical stimulation of aerial layering for other species in less fire-prone ecosystems or for horticultural purposes (Hartmann and Kester [1975]; O’Dea et al. [1995]; Lantz and Antos [2002]; Deb and Pogener [2012]). As woody plants continue to encroach and transition these critical areas for conservation from grasslands to shrublands, it is increasingly critical that we continue to understand the organismal communities and ecological drivers (Samson [2004]; Briggs et al. [2005]; Zhang and Zhang [2007]). We suggest that aerial layering be considered as an adaptation to fire disturbances and that additional research is warranted. Areas for additional inquiry include how aerial layering and seed production of *S. orbiculatus* fluctuate along a gradient of disturbance and how fire intensity and fire return interval affect belowground total non-structural carbohydrate reserves because modern anthropogenic fire regimes may not replicate a natural lighting-ignited fire regime (Kennedy and Potgieter [2003]; de Groot and Wein [2004]). Finally, it is also important to understand how this basic plant ecology information applies to other resprouting shrub species that use a layering mechanism and how spatially heterogeneous prescribed burning functions in land management and conservation (Hodgkinson [1998]; Doumas and Koprowski [2012]).

## Author’s information

JDS conducted the sampling portion of this project while a graduate student at Oklahoma State University.

## Authors’ original submitted files for images

Below are the links to the authors’ original submitted files for images. Authors’ original file for figure 1 Authors’ original file for figure 2 Authors’ original file for figure 3

## Abbreviations

AUM: Animal unit month
GAB: Graze and burn treatment
GRG: Grand River Grasslands
NOAA: National Oceanic, and Atmospheric Administration
PBG: Patch-burn grazing treatment
PDSI: Palmer’s Drought Severity Index
USDA: United States Department of Agriculture
