# Supplementary figures and images for: Fire induced reproductive mechanisms of a Symphoricarpos (Caprifoliaceae) shrub after dormant season burning
Source: Bot Stud. 2014 Dec 24;55:80. doi: 10.1186/s40529-014-0080-4 (PMC5432769; doi:10.1186/s40529-014-0080-4)

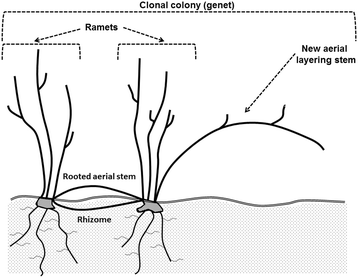

Supplement: Supplementary file 1 — Authors’ original file for figure 1 [file 40529_2014_9080_MOESM1_ESM.gif]

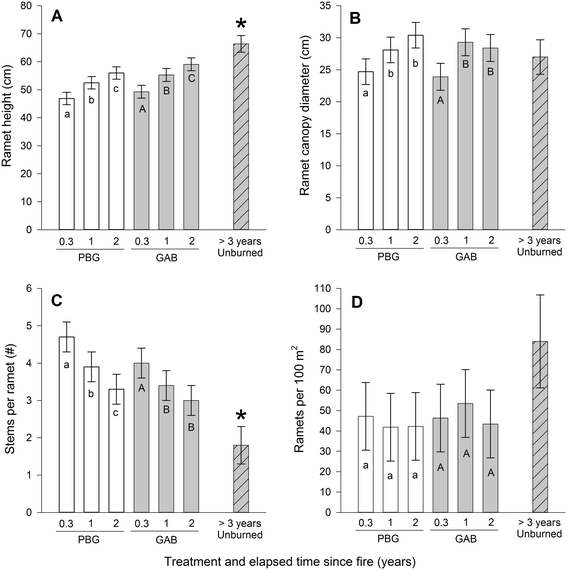

Supplement: Supplementary file 2 — Authors’ original file for figure 2 [file 40529_2014_9080_MOESM2_ESM.gif]

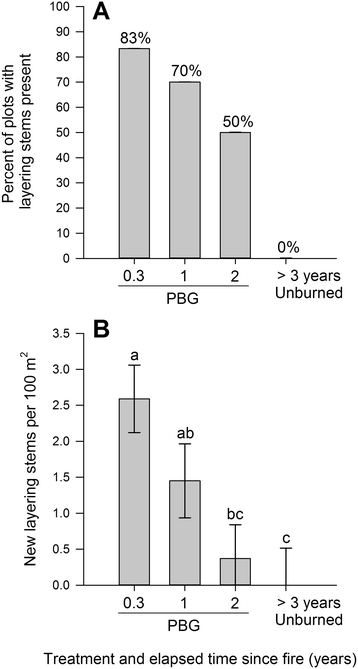

Supplement: Supplementary file 3 — Authors’ original file for figure 3 [file 40529_2014_9080_MOESM3_ESM.gif]
